# Glycans of SARS-CoV-2 Spike Protein in Virus Infection and Antibody Production

**DOI:** 10.3389/fmolb.2021.629873

**Published:** 2021-04-13

**Authors:** Xiaohui Zhao, Huan Chen, Hongliang Wang

**Affiliations:** Department of Pathogen Biology and Immunology, Xi’an Jiaotong University Health Science Center, Xi’an, China

**Keywords:** SARS-CoV-2 (2019-nCoV), vaccine, glycosylation, structure, viral entry

## Abstract

Viral protein glycosylation represents a successful strategy employed by the parasite to take advantage of host–cell machinery for modification of its own proteins. The resulting glycans have unneglectable roles in viral infection and immune response. The spike (S) protein of severe acute respiratory syndrome coronavirus 2 (SARS-CoV-2), which presents on the surface of matured virion and mediates viral entry into the host, also undergoes extensive glycosylation to shield it from the human defense system. It is believed that the ongoing COVID-19 pandemic with more than 90,000,000 infections and 1,900,000 deaths is partly due to its successful glycosylation strategy. On the other hand, while glycan patches on S protein have been reported to shield the host immune response by masking “nonself” viral peptides with “self-glycans,” the epitopes are also important in eliciting neutralizing antibodies. In this review, we will summarize the roles of S protein glycans in mediating virus–receptor interactions, and in antibody production, as well as indications for vaccine development.

## Introduction

Since its first emergence in December 2019, it only took several months before the novel coronavirus disease 2019 (COVID-19), a severe respiratory illness, was declared as a pandemic by the World Health Organization (https://www.who.int/docs/default-source/coronaviruse/situation-reports/20200311-sitrep-51-covid-19.pdf?sfvrsn=1ba62e57_10). The causative agent was identified to be a member of *Betacoronavirus* and termed as SARS-CoV-2 (Coronaviridae Study Group of the International Committee on Taxonomy of Viruses, 2020). Coronaviruses (CoVs) are enveloped positive-sense RNA viruses, and as a virus with an RNA genome, CoVs have high mutation rates and hence are believed to alter host range and tissue tropism efficiently (Li, 2016; Cui et al., 2019; Hu et al., 2020). CoVs are responsible for multiple respiratory disorders of varying severity in humans (Cui et al., 2019). Seven coronavirus strains are known to cause human infection; among them, HCoV 229E, HCoV NL63, HCoV HKU1, and HCoV OC43 typically cause only mild upper respiratory diseases in immunocompetent hosts, although some of them can cause severe infections in infants, young children, and elderly individuals (Cui et al., 2019), while severe acute respiratory syndrome coronavirus (SARS-CoV), Middle East respiratory syndrome coronavirus (MERS-CoV), and SARS-CoV-2 cause severe respiratory illness and fatalities (Cui et al., 2019; Hasöksüz et al., 2020).

The spike protein (S) of coronavirus, which forms large protrusions from the virus surface and gives the virus the appearance of having crowns, mediates virus entry into host cells (Hu et al., 2020; Cui et al., 2019; Hasöksüz et al., 2020; Virology, 1968). Therefore, the S protein is a critical determinant of viral host and tissue tropism. In addition, the S protein is glycosylated by the host cellular glycosylation apparatus as it passes through the secretory pathway. These glycans confer two benefits on the virus. First, the mannose residues within these glycans are important moieties to interact with cell surface attachment factors, like glycosaminoglycans (GAGs) and sialic acid-containing oligosaccharides (Li et al., 2017; Tortorici et al., 2019; Robson, 2020), before binding to the high-affinity receptor—in the case of SARS-CoV-2, angiotensin-converting enzyme 2 (ACE2) (Hoffmann et al., 2020; Zhou et al., 2020). In the complex of spike–ACE2, extensive glycosylation at the interface of the complex was reported (Zhao et al., 2020), highlighting roles for glycans in modulating spike–ACE2 interactions. Second, glycans sterically mask the underlying polypeptide epitopes from recognition of potentially neutralizing antibodies, and thus sometimes referred to as the “glycan shield” (Doores, 2015; Bagdonaite and Wandall, 2018). Viral glycoproteins are the main targets of host antibodies, as these molecules are prominently displayed on the virion surfaces (Murin et al., 2019). Different from bacteria, in which glycans are encoded by the bacterial genome and are treated as “nonself” epitopes by corresponding hosts, viruses take advantage of host cell machinery for glycosylation and generally are decorated with the “self”-glycans. These “self”-glycans are generally thought to be a strategy to escape the host immune response (Wang, 2020). For example, human immunodeficiency virus (HIV-1) (Stewart-Jones et al., 2016), hepatitis C virus (Falkowska et al., 2007), and Ebola virus (Iraqi et al., 2020) exhibit extensive N-linked glycans that cover some of the critical virus-neutralizing epitopes to block antibody recognition. Similarly, coronavirus S glycans also mask the protein surface and consequently limit antibody access to protein-neutralizing epitopes (Grant et al., 2020; Wang, 2020; Watanabe et al., 2020). Therefore, understanding the glycosylation of S protein has important implications in viral pathobiology and vaccine design.

In addition to S protein, glycosylation of E protein, M protein, and nonstructural proteins in SARS-CoV has also been predicted, and their potential roles are discussed in a review (Fung Liu, 2018). In this review, we will mainly focus on the glycosylation of S protein.

### Spike Protein and Glycosylation

**FIGURE 1:**
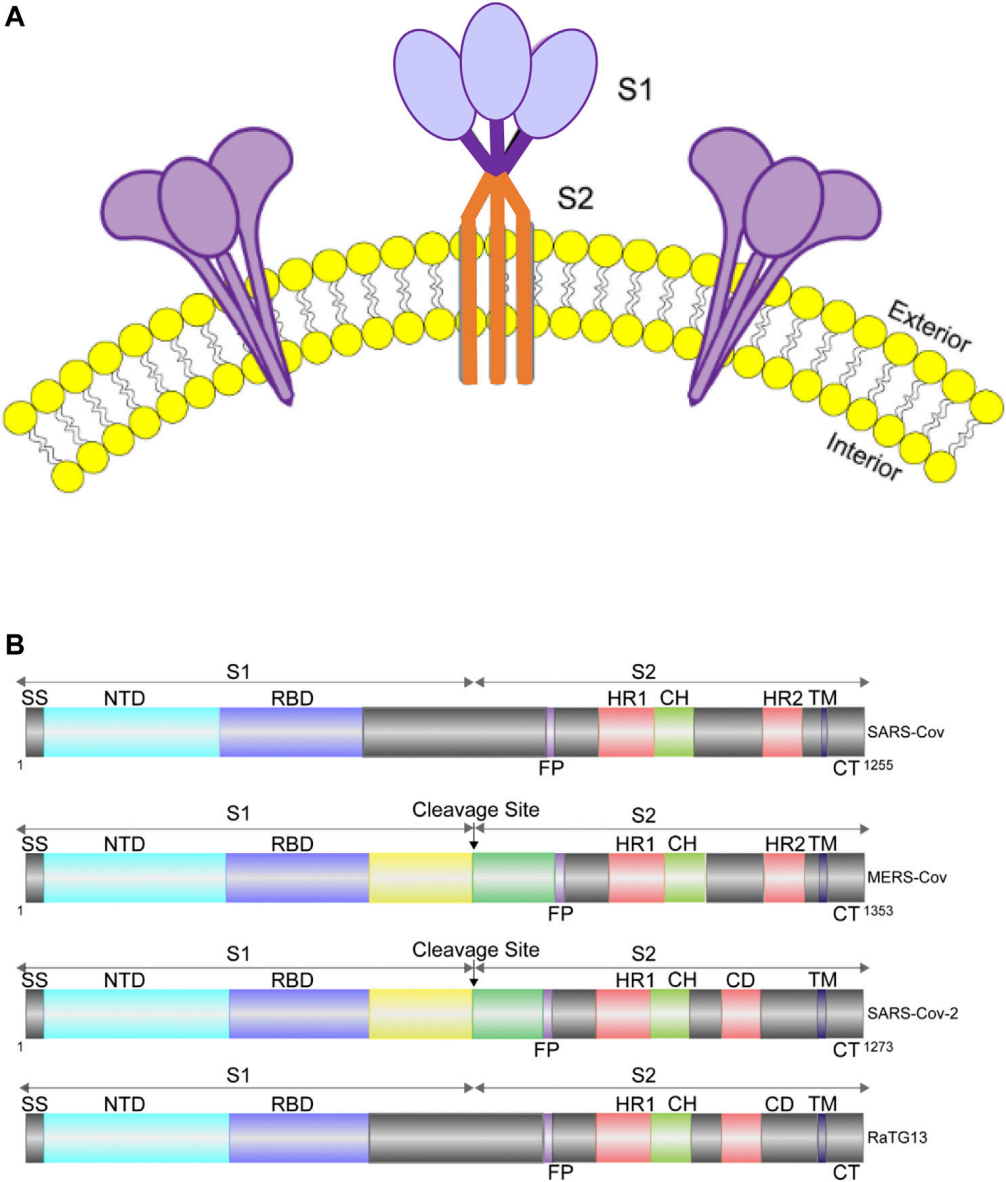
Cartoon representation of S protein structures. **(A)**. Diagram of S protein trimers on viral envelope (yellow: lipid bilayer). **(B)**. Diagram showing the domain organization of the S proteins of SARS-CoV, MERS-CoV, RaTG13 and SARS-CoV-2. MERS-CoV and SARS-CoV-2 can be cleaved into S1 and S2 by cellular protease, while SARS-CoV and RaTG13 cannot. SS, signal sequence; RBD, receptor-binding domain; FP, fusion peptide; HR1, heptad repeat 1; CH, central helix; CD, connector domain; HR2, heptad repeat 2; TM, transmembrane domain; CT, cytoplasmic tail.

The S protein, which is conserved to various degrees across the Coronaviridae family, is the most critical structural protein of SARS-CoV-2. It forms homotrimers and protrudes from the viral surface (Figure 1A), which makes the virus reminiscent of the solar corona, and it plays a key role in the virus entry into the host cell (Hasöksüz et al., 2020; Virology, 1968; Wrapp et al., 2020).

The S protein is a type I membrane protein, synthesized in the endoplasmic reticulum, and is transported to the plasma membrane (Bosch et al., 2003). For SARS-CoV-2, the total length of S is 1,273 amino acids and consists of a signal peptide located at the N-terminus. It has been reported that S of SARS-CoV-2 contains a furin cleavage site, which contains multiple basic amino acids (PRRAR) and can be cleaved into two functional subunits: S1 subunit, which is responsible for binding to cellular receptor; and S2 subunit, which functions to fuse the viral and cellular membranes (Hoffmann et al., 2020; Walls et al., 2020; Xia et al., 2020). After cleavage, both subunits remain non-covalently bound in the prefusion conformation (Wrapp et al., 2020; Walls et al., 2020; Kirchdoerfer et al., 2016). In contrast, S of SARS-CoV and other SARS-related coronaviruses (SARSr-CoVs), particularly RaTG13, which has 96% identity of its genomic sequence to that of SARS-CoV-2, does not contain a furin-like motif (Xia et al., 2020) (Figure 1B). The function of this cleavage site is being extensively studied (Johnson et al., 2020; Walls et al., 2020; Xia et al., 2020).

**FIGURE 2:**
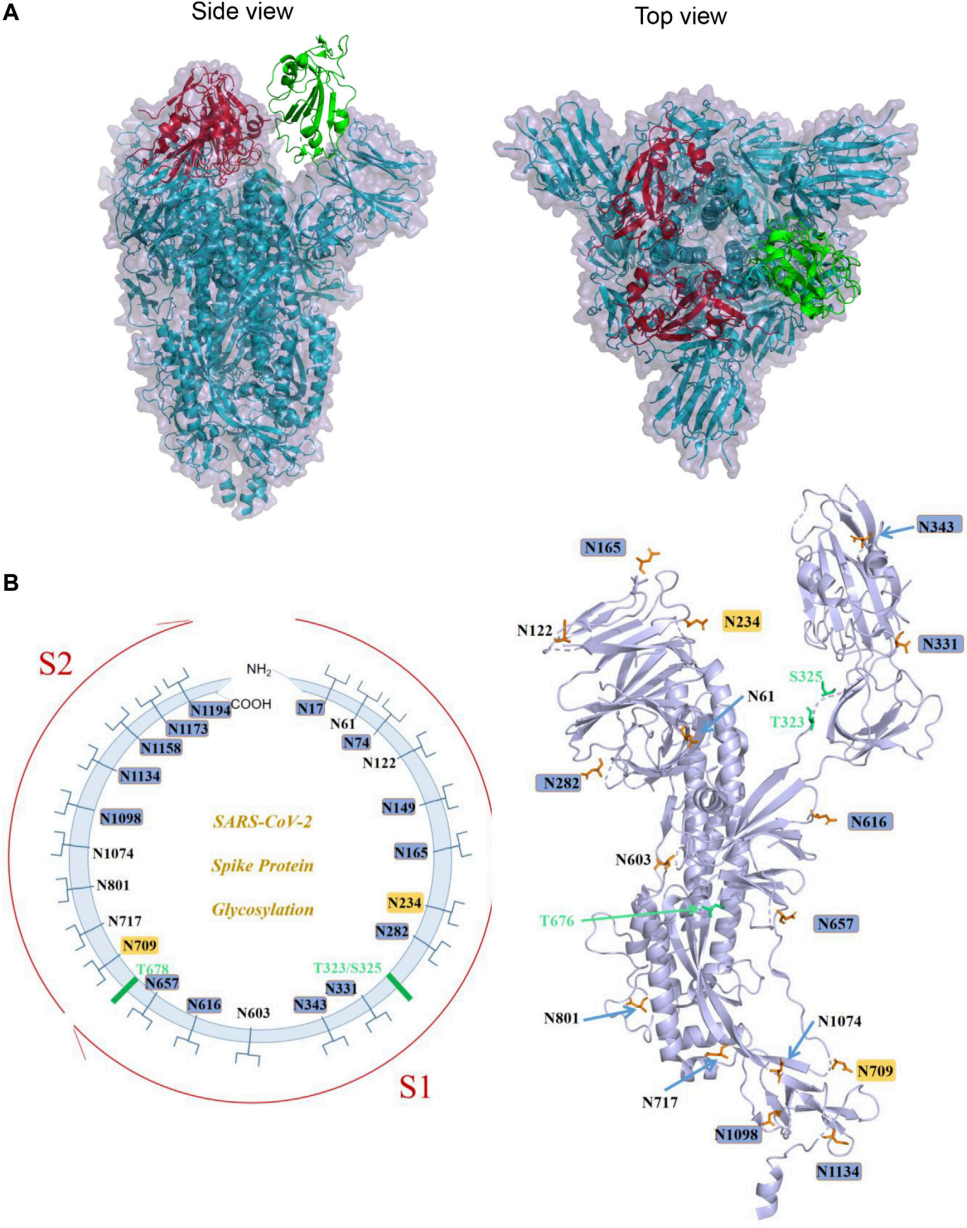
S protein structure of SARS-CoV-2 and its known glycosylation. **(A)**. The trimeric structure of S protein ectodomain (PDB ID: 6VYB): two red RBDs are in the “down” conformation, and the green one is in “up” conformation (Left panel: side view; right panel: top view). **(B)**. Left panel: schematic diagram demonstrating the known sites of glycosylation on the S glycoprotein. N-linked glycosylations are shown as branches. The most processed glycans are highlighted in blue, and the least processed are highlighted in yellow. The positions of O-linked glycosylation sequons are shown as green bars. Right panel: A spatial representation of known glycosylation on S protein monomeric structure demonstrating the locations of N- and O-glycosylation. Image generated by Pymol based on PDB ID 6VXX.

Structures of S from other *Betacoronavirus* have shown that the receptor-binding domain (RBD) of S1 undergoes hinge-like conformational movements that transiently hide or expose the determinants of receptor binding (Gui et al., 2017; Pallesen et al., 2017; Yuan et al., 2017). The “closed” conformation is three-fold symmetric and has all three RBDs in “down” positions. In this conformation, RBD is not receptor-accessible due to steric clashes, while the “open” conformation is asymmetric, in which at least one RBD is in an “up” position and is used for receptor binding (Gui et al., 2017; Yuan et al., 2017). The 3D structure of SARS-CoV-2 S trimer in the prefusion conformation has also been determined by cryo-EM (Walls et al., 2020; Wrapp et al., 2020). Overall, the SARS-CoV-2 S ectodomain is a 160-A°-long trimer with a triangular cross section (Walls et al., 2020). And similar to SARS-CoV S, a single RBD was observed in the “up” position in the prefusion state for receptor binding, with the other two RBDs in the “down” position (Walls et al., 2020; Wrapp et al., 2020; Figure 2A).

Another key feature of the S protein is its extensive glycosylation which depends on the host glycosylation machinery. Depending on the acceptor amino acid atom to which the sugar moiety is attached, glycosylation can be divided into N-linked glycosylation (sugars are attached to the amide nitrogen atom in the side chain of asparagine) and O-linked glycosylation (sugars are attached to the oxygen atom in the side chain of serine or threonine) (Berg et al., 2002; Bagdonaite and Wandall, 2018). An asparagine residue can accept an oligosaccharide only if the residue is part of an Asn-X-Ser or Asn-X-Thr sequence, in which X can be any residue except proline (Breitling and Aebi, 2013). In contrast, there are no conserved protein sequence motifs for general or isoform-specific O-glycosylation, and therefore it is much more difficult to predict this modification (Gerken et al., 2011; Bagdonaite and Wandall, 2018). O-glycans have also been observed on some viral proteins and have been suggested to play roles in the biological activity of viral proteins (Bagdonaite and Wandall, 2018; Andersen et al., 2020).

The monomer of the SARS-CoV-2 S protein comprises 22 N-linked glycosylation sequons. Walls et al. first resolved 16 of them with oligosaccharides with cryo-EM (Walls et al., 2020). Later on, by using a site-specific mass spectrometric (MS) approach, Watanabe et al. proved that all sites are glycosylated, and they further analyzed the glycosylation pattern of these 22 sites and found that two sites on SARS-CoV-2 S are principally oligomannose-type (least processed): N234 and N709. A mixture of oligomannose- and complex-type (most processed) glycans can be found at sites N61, N122, N603, N717, N801, and N1074, while the remaining sits have complex glycans (Virology, 1968) (Figure 2B). Zhao et al. also reported the site-specific N-linked microheterogeneity with similar results and suggested that these glycans sterically masked polypeptide epitopes and directly modulated spike–ACE2 interactions (Zhao et al., 2020).

In addition to the MS-based approaches that investigate the global glycoprofile of the protein, Lenza et al. characterized the glycan structures of N-linked glycan in the RBD using nuclear magnetic resonance (NMR) spectroscopy (Lenza et al., 2020). Compared to MS, NMR avoids sample digestions, thus providing glycan information of the intact protein in a physiological-like solution. For example, the importance of N-acetylgalactosylation and hyper-fucosylation at the terminal chains of the RBD N-glycans was revealed, and novel glycan motifs, such as 4SulLDN, 6′SLDN, LeX, and LDNF, were identified by the NMR-based study (Lenza et al., 2020).

In contrast to the consistent results of N-linked glycosylation, different groups have reported different O-linked glycosylation patterns of S protein depending on different protein expression systems and detection methods employed. Shajahan et al. reported a high level of O-glycosylation of S1 and S2 when expressed independently, and detected O-glycosylation at sites Thr323 and Ser325 on the S1 subunit of the S protein (Shajahan et al., 2020). However, other two reports detected low occupancy at most sites of O-glycan modification using S trimer for analysis (Watanabe et al., 2020; Zhao et al., 2020). One possible explanation for this discrepancy is that the S protein could undergo different types of glycosylation at different conformations or oligomeric states. In addition, Andersen et al. predicted a unique O-linked glycosylation pattern flanking the furin cleavage site (Andersen et al., 2020), and glycosylation around this cleavage site is thought to regulate the activation of the S protein. Sanda et al. confirmed this O-glycosylation near the furin cleavage site (T678) using MS-based methods; in addition, they identified another eight O-glycopeptides (Sanda et al., 2020). The functional role of most of the O-linked glycosylation is not fully understood.

### Glycans in Viral Entry

Glycans on viral envelope glycoproteins play important roles in virus infection, with specific functions identified in various stages of viral infection (Bagdonaite and Wandall, 2018). But, in general, most of the function falls into the stage of viral entry and release. To date, as few studies have been conducted on the role of glycans in SARS-CoV-2 release; here in this review, we will focus on their roles in viral entry. Viral entry into host cells is initiated by the interaction of virion with cell surface attachment factors. And host cell glycans often serve as the front lines of initial contacts to facilitate following high-affinity binding to virus-specific receptors (Watanabe et al., 2019; Koehler et al., 2020). In addition, some cell surface glycans also function as co-receptors or specific entry receptors (Raman et al., 2016). Therefore, glycans from both the viral glycoprotein and host cell surface are involved in viral attachment and are critical for efficient viral entry.

Similarly, SARS-CoV-2 also exploited this low-to-high-affinity strategy for efficient viral attachment and entry. S protein was able to bind heparan sulfate or sialic acids to initiate the attachment (Baker et al., 2020; Hao et al., 2020; Liu et al., 2020). On the other hand, many coronaviruses have a galectin-like domain in the N-terminal domain (NTD) of S1, which functions as viral lectins to bind cell surface sugars, like 5-*N*-acetyl-9-*O*-acetylneuraminic acid (Peng et al., 2011; Peng et al., 2012). Meanwhile, a similar ganglioside-binding domain is found in the NTD of SARS-CoV-2 S protein. In the spike trimers, the RBD locates at the center, while the NTD lies laterally, enabling the ganglioside-binding domain to interact with the large area of lipid rafts on cell surface (Fantini et al., 2020). Together, these low-affinity interactions may facilitate virus binding to the high-affinity receptor ACE2.

**FIGURE 3:**
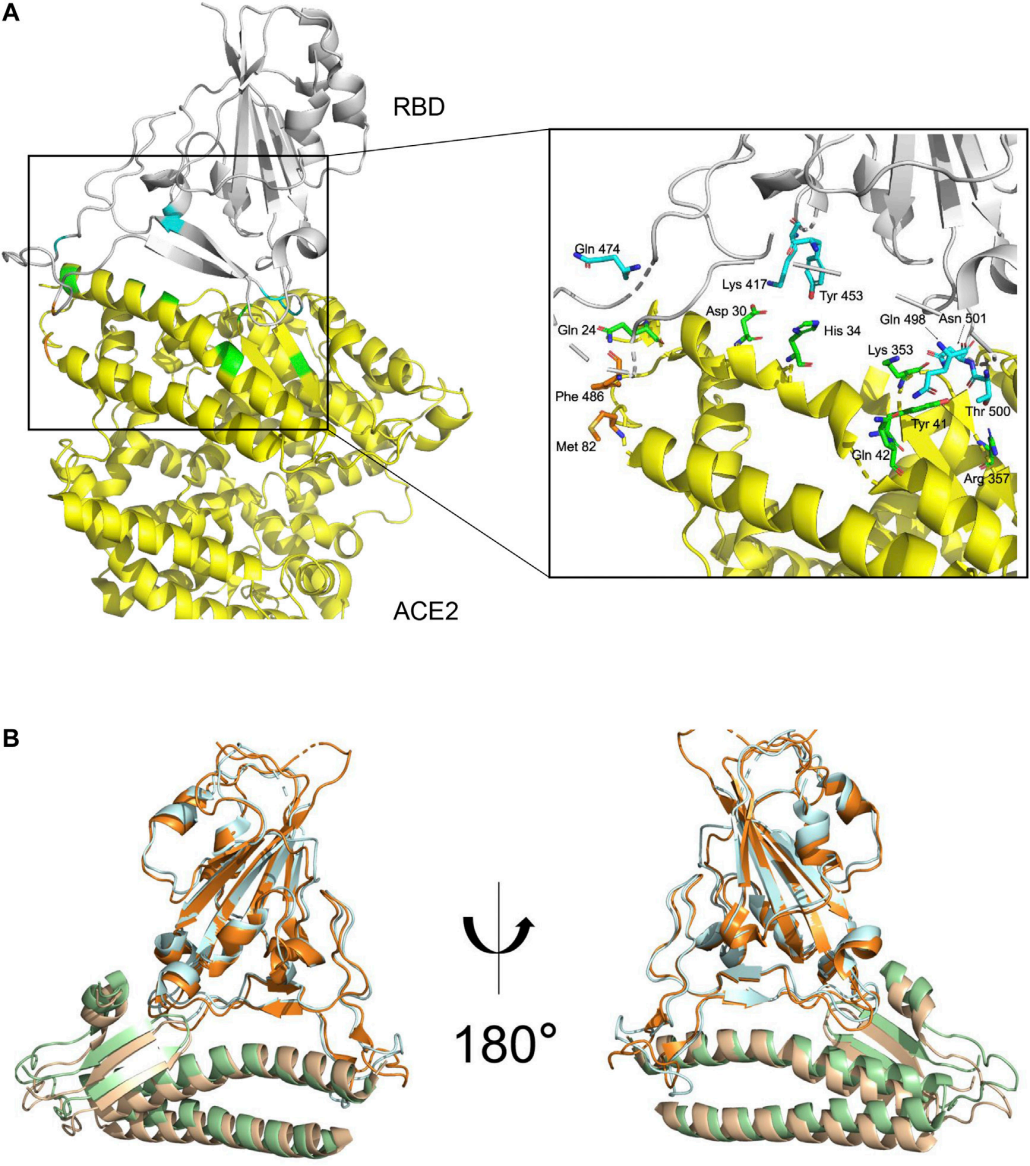
S protein and ACE2 receptor interaction. **(A)**. The complex structure of RBD–ACE2 demonstrating the interactions between SARS-CoV-2 RBD (gray) and ACE2 (yellow) with key residues on RBD and ectodomain of ACE2 shown in blue and green, respectively. Images generated based on PDB 6M0J. **(B)**. Structural superimpose of the SARS-CoV-2-RBD/ACE2 and SARS-CoV-RBD/ACE2 complexes. SARS-CoV-RBD and ectodomain of ACE2 are colored in cyan and green, respectively. SARS-CoV interactions were generated based on PDB 2AJF.

The structure of the complex of ACE2 and RBD of S has recently been determined by cryo-EM (Yan et al., 2020) and X-ray crystallography (Lan et al., 2020; Figure 3A). The overall complex structure showed that ACE2 has two lobes, and RBD contacts the bottom side of the small lobe of ACE2. The key residues in the SARS-CoV-2 RBD and ACE2 binding are highly conserved or share similar side chain properties with those in the SARS-CoV RBD (Yan et al., 2020; Lan et al., 2020; Figure 3B). In addition, the cryo-EM results also suggested that ACE2 formed heterodimer, in which they might simultaneously bind to two S protein trimers (Lan et al., 2020).

**FIGURE 4:**
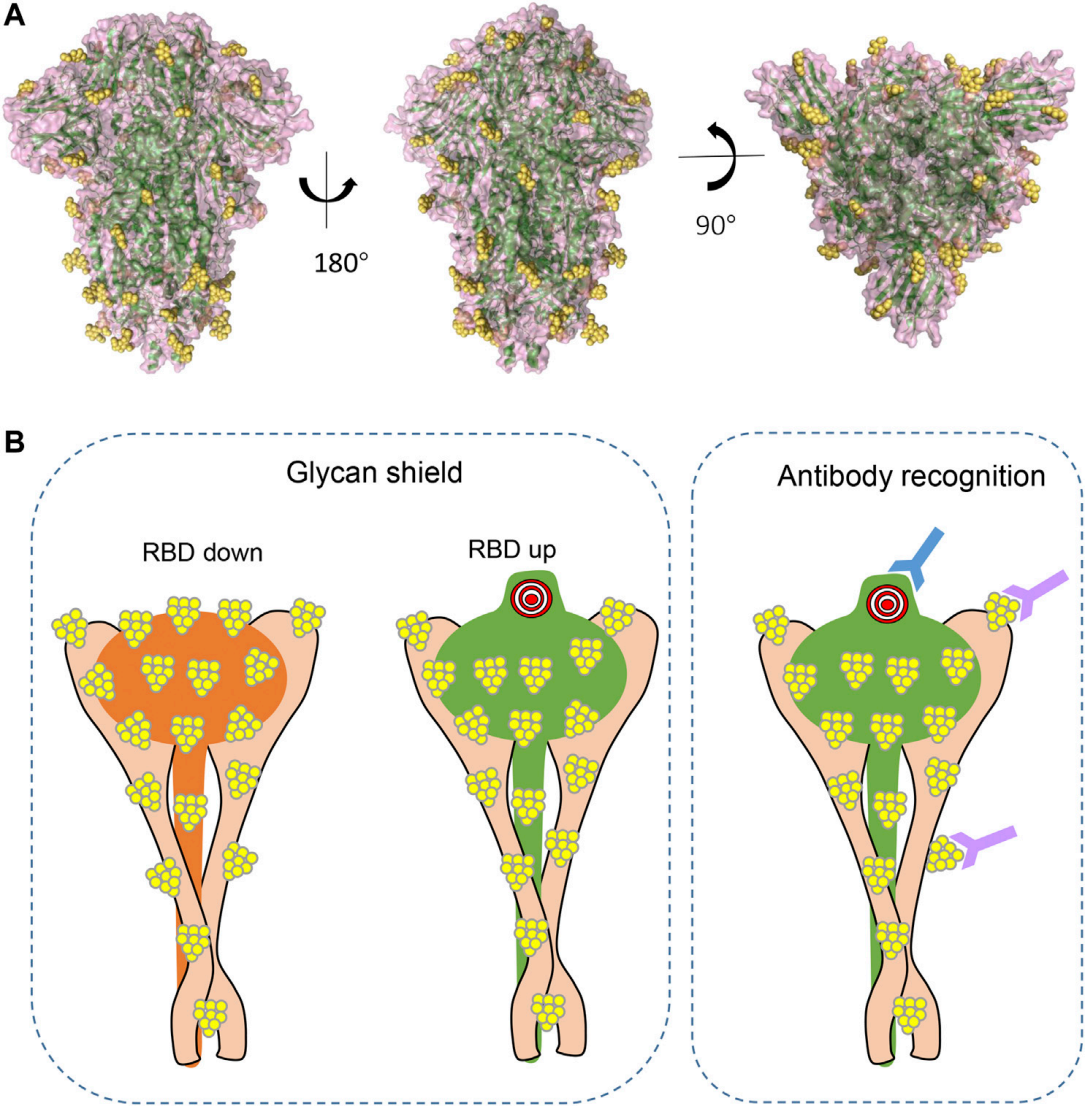
The shielding effect of glycosylation on the S protein trimer **(A)**. Structural representations from different views demonstrating glycan epitopes. Yellow balls represent glycans, and S backbone was generated based on PDB ID: 6VXX. **(B)**. Diagram of glycans in molecular recognition shielding and their potential role in antibody elicitation (left panel: compared to RBD in “up” position, RBD in “down” position has larger coverage of glycan; right panel: in addition to peptide epitopes, some glycans can also be recognized by antibody and are thus important epitopes in antibody production).

Extensive glycosylation has also been characterized at the interface of S and ACE2 interaction (Zhao et al., 2020; Watanabe et al., 2020; Casalino et al., 2020). As mentioned earlier, glycans of viral surface protein may shield the protein from molecular recognition (Figure 4A). However, to effectively function, the S RBD region must be fully exposed and accessible to bind ACE2 receptor to initiate infection. By using all-atom molecular dynamic simulation (MD), two N-glycan sites adjacent to the RBD (N165 and N234) were predicted to function at the interface of ACE2 interaction (Casalino et al., 2020). Further analysis showed that the “down” conformation of RBD has a remarkably larger coverage of glycans than that of RBD in “up” conformations (Casalino et al., 2020), indicating the virus has evolved mechanisms for receptor recognition and effective infection (Figure 4B, left panel). Unexpectedly, N165A and N234A mutations, which led to glycan deletions at the respective sites, were shown to have reduced binding to ACE2 receptor (Casalino et al., 2020), suggesting that these glycans are actually important for the conformational plasticity of the RBD and hence ACE2 interaction. Consistent with this, Watanabe et al. also observed glycosylation on these sites (Watanabe et al., 2020), and this shielding of receptor binding sites by glycans is a common feature of viral glycoproteins, as observed on other viruses (Zhao et al., 2020). In addition, glycans on ACE2 molecules are also important for the virus–receptor interaction. For example, two glycans on ACE2 (at N090 and N322) can form interactions with the S protein, of which the N322 glycan interaction with the S trimer is outside of the RBD, while the N090 glycan is close to the S trimer surface (Zhao et al., 2020). Non-synonymous single-nucleotide polymorphisms (SNPs) in the ACE2 glycosylation site have been reported in the human population and proposed to alter ACE2–S protein interaction (Zhao et al., 2020), and thus might affect the viral infection progress and severity of the disease. Last, intermolecular glycan–glycan interactions are also observed between the glycans of ACE2 and those in the S protein (Zhao et al., 2020). These glycan-mediated interactions are vital for efficient viral infection.

### Glycans in Antibody Production

During the battle against pathogen infections, our immune system has developed sophisticated mechanisms to identify and eliminate invading pathogens, including viruses. In turn, viruses have also evolved mechanisms to counteract or take advantage of the host’s defense mechanisms (Finlay and McFadden, 2006; Rouse and Sehrawat, 2010). Glycans on viral glycoproteins have dual roles in virus–host interplay. On the one hand, glycans can be part of the immune determinants themselves, but on the other hand, glycans can mask the antigenic protein epitopes from recognition (Bagdonaite and Wandall, 2018). For most CoVs, S is virtually the only antigen present at the virus surface, it is the major target for host antibodies, and this viral antigen is therefore a main target for vaccine development (Conte et al., 2020). A comprehensive SARS-CoV-2 S protein immunogen-induced antibody signature study found that although S, S1, S2, and RBD all can elicit antibody, RBD immunogen elicited a higher antibody titer with higher affinity (Ravichandran et al., 2020), this is in consistent with the fact that RBD is the most exposed epitope of S (Grant et al., 2020). And due to the sequence conservation, one might expect SARS-CoV S to elicit cross-reactive antibody against SARS-CoV-2 (Ou et al., 2020; Pinto et al., 2020; Walls et al., 2020). However, certain existing monoclonal antibodies raised against the SARS-CoV RBD (S230, m396, and 80R) failed to bind the RBD of SARS-CoV-2 S (Lan et al., 2020; Wrapp et al., 2020), reflecting a divergence of antigenic epitopes among different CoVs.

#### Glycan as Shield

As S is highly glycosylated, one would predict that glycan shielding is important in immune evasion. MD simulations predicted that S antigen was covered with glycans that will shield antibody recognition (Figures 4A,B left panel). Depending on the glycoforms, the exposed surface area is between 53 and 71%, and the most exposed epitopes, as expected, comprise the RBD that interacts with ACE2 (Grant et al., 2020). Compared to S proteins of MERS-CoV and SARS-CoV, and viral envelope glycoproteins of HIV-1, SARS-CoV-2 S is less densely glycosylated (Behrens et al., 2016; Walls et al., 2019; Grant et al., 2020; Watanabe et al., 2020), suggesting the protein surface is more exposed and may elicit humoral immunity more efficiently. Beyond a role in shielding the underlying protein from recognition by antibodies, the glycans may also attenuate the T-cell response. For example, it has been reported that some glycans in S may interfere with antigen presentation in an HLA complex (Grant et al., 2020).

#### Glycan as Epitopes

The high density of glycans creates a shield that blocks antibody recognition, but some of the most potent and broadly neutralizing antibodies are able to recognize these glycan epitopes (Doores, 2015) (Figure 4B, right panel). A recent study has shown that glycan clusters on viral proteins are different from those of host protein glycosylation (Wang et al., 2015), making them good candidates for immune response induction. In fact, 2G12, isolated from the serum of an HIV-positive individual, was the first reported broad neutralizing antibody to bind to carbohydrates (α1->2 mannose) on HIV gp120 (Buchacher et al., 1994; Trkola et al., 1996; Scanlan et al., 2002). In addition to these mannose patch–targeted antibodies, antibodies recognizing complex-type glycans have also been reported against HIV glycoprotein (Mouquet et al., 2012; Pancera et al., 2013; Falkowska et al., 2014). In addition, broadly neutralizing antibodies with epitopes comprising glycans have also been characterized against dengue virus E protein (Rouvinski et al., 2015), all these suggesting that glycans are important moieties to elicit broadly neutralizing antibodies. The SARS-CoV-2-S comprises 22 N-linked glycosylation sites with different glycoforms, and some of them may also elicit neutralizing antibodies. In fact, a recent study showed that one antibody, that is, S309, which potently neutralizes both SARS-CoV and SARS-CoV-2, actually recognizes an epitope containing a glycan that is conserved within the Sarbecovirus subgenus (Pinto et al., 2020), suggesting that glycans in SARS-CoV-2 are important in neutralizing antibody production. Whether other glycans are antigenic and could elicit neutralizing antibodies need further investigation.

#### Glycans and Autoimmunity

Glycans in the glycoprotein could stimulate the production of carbohydrate-specific antibodies, which play important roles in protecting the host from infectious disease. On the other hand, they might also cross-react with host antigens, leading to autoimmune diseases (Patel and Kearney, 2016; Kappler and Hennet, 2020). Previous studies have demonstrated that autoimmune diseases, like Guillain–Barre syndrome, multiple sclerosis, and inflammatory bowel disease, are caused by carbohydrate-specific antibodies related to bacterial infection or gut microbes (Kappler and Hennet, 2020). A recent SARS-CoV-2 study showed that COVID-19 patients have unusually high IgM and IgG antibodies to self-carbohydrates, including gangliosides, *N*-linked glycans, LacNAc derivatives, blood group H1, and sialyl Lewis X (Butler and Gildersleeve, 2020), indicating the possibility of autoimmune disorders caused by these antibodies. Noteworthily, many of the glycan antibodies are naturally occurring antibodies and are detectable in the absence of deliberate immunization or vaccination (Kappler and Hennet, 2020), and these antibodies are low in affinity and specificity; therefore, their roles in SARS-CoV-2 patient autoimmunity remain unclear.

### Glycans in Vaccine Development

Many efforts have been directed toward the development of the vaccines against SARS-CoV-2. Due to its high antigenicity and ability to elicit neutralizing antibodies in convalescent individuals (Cao et al., 2020; Ju et al., 2020), S protein is an ideal candidate for vaccine development. Currently, dozens of vaccine candidates based on S or its subunits are in rapid development (Sternberg and Naujokat, 2020), be it delivered by the recombinant protein, DNA, mRNA, or viral vector–based form. Among these vaccine types, mRNA vaccines have shown great potential and offered advantages over conventional vaccines. A recent study showed that a single immunization of SARS-CoV-2 mRNA vaccine elicited potent germinal center responses and neutralizing antibody production, in contrast to the poor performance of recombinant protein vaccine (Lederer et al., 2020). The fact that different forms of S protein would have different glycosylation patterns (Brun et al., 2020; Watanabe et al., 2020) suggests that nucleic acid–based vaccines may produce epitopes with different glycosylations from other types of vaccine, resulting in different immune responses. As it is widely accepted that the lack of information about protein glycosylation hampers the design or compromises the efficacy of vaccines (Wolfert and Boons, 2013), glycosylation of S protein should be taken into consideration in the development of SARS-CoV-2 vaccines.

Glycans have been well considered in developing HIV broadly neutralizing antibodies (Duan et al., 2018; Escolano et al., 2019; Watanabe et al., 2019; Seabright et al., 2020). By introducing N-linked glycans into non–CD4-binding site surfaces to mask irrelevant epitopes, higher proportions of CD4-binding site–specific antibody were produced (Duan et al., 2018), suggesting glycan masking can be used to limit off-target antibody production and increase on-target immune response. Escolano et al. designed an immunogen that recognized the V3-glycan patch on the envelope protein of HIV-1 and found this immunogen elicited specific serological responses in mice or macaques (Escolano et al., 2019). In reviewing the current SARS-CoV-2 vaccine candidates, most of them are based on S peptide epitopes, and glycans are rarely been considered. However, awareness is increasing, and more studies will address the need of relevant glycosylation in SARS-CoV-2 vaccine design. In fact, since studies on inactivated influenza virus and recombinant gp120 of HIV vaccines indicate that glycoengineering of glycan shields to present α-gal epitopes enables antibody for amplifying vaccine efficacy, glycoengineering of α-gal epitopes on SARS-CoV-2 vaccines has been proposed (Galili, 2020). Given the fact that antibody S309 neutralizes both SARS-CoV and SARS-CoV-2, glycan-based vaccine or broad neutralizing antibody against closely related CoVs is promising.

### Future Perspectives

It has long been known that glycosylation of viral envelope proteins is essential for infectivity and affects immune recognition. Here, we briefly summarized the roles of SARS-CoV-2 S protein glycans in viral infection and antibody elicitation. With rapidly evolving analytical methods toward glycan determination and increasing awareness of the importance of glycans in virus biology and vaccine design, we will have a complete picture of virus glycosylation, and their roles in viral infection and host immune response.
